# Development of a social skills training programme to target social isolation using virtual reality technology in primary mental health care

**DOI:** 10.1177/20556683231187545

**Published:** 2023-07-11

**Authors:** Solveig Osborg Ose, Kristin Thaulow, Hilde Færevik, Per Lund Hoffmann, Hedvig Lestander, Tore Stiles, Martin Lindgren

**Affiliations:** 1 63283 SINTEF Digital, Trondheim, Norway; 2 63283 Trondheim Municipality, Norway; 3 Coperio AS, Trondheim, Norway

**Keywords:** Virtual reality technology, psychiatric rehabilitation, primary mental health services, social skill training, social isolation

## Abstract

**Introduction:**

People with severe mental illness often have a small or no network of friends and limited contact with their family and live social isolated lives. We developed a social skills training programme to be administered by public mental health professionals in helping those with mental illness to overcome their social isolation.

**Methods:**

The programme was developed over 3 years in close collaboration among psychologists, service users, municipal mental health professionals, mental health service researchers and a local firm providing virtual reality (VR) training. We started with the simplest available equipment, that is, a cardboard headset combined with a smartphone, then we used Oculus Quest and now Oculus Quest 2.

**Results:**

The resulting programme is comprised of eight steps from: 1) identify service user’s primary and secondary goals to 8) three-month follow-up.

**Conclusion:**

Several factors made adoption and implementation of VR technology possible in a relatively short timeframe: namely, the municipality and service users were involved from the beginning of the development process, efforts were made to introduce VR to mental health professionals and allow them to reflect on its usability, solutions were low-tech and low cost, and the long-term research collaboration was established without municipal financial obligations.

## Introduction

In recent decades, adult mental health services in Norway have been characterised by deinstitutionalisation and redistribution of patients from psychiatric hospitals to decentralised services,^1^ which is similar to mental health care approaches in most industrialised countries.^2^ This shift is based on the assertion that most care should be provided at or near service users’ homes.^3^ Primary mental healthcare services in the municipalities are thus increasingly expected to provide psychiatric rehabilitation including training in social skills to reduce social isolation.^4^

Mental health services currently lack effective tools to reduce social isolation among both primary and specialist service users. Those living socially isolated lives with severe mental health problems often experience multiple barriers to attending low-threshold services and other available local services. Social interactions are so difficult for some service users that they rarely leave their residence. Activities that are usual for most people, such as visiting the grocery store or gym, or having a coffee at a café, are insurmountable to many mental health service users. Thus, new methods are needed to help those who self-isolate from fear, lack of self-confidence or thoughts that compel them to stay isolated and lonely. Municipal mental health service employees offer some assistance to service users, such as going with them to the grocery store. However, this does not necessarily improve service users’ functioning or reduce barriers to visiting the store independently; therefore, service users’ needs for ongoing accompaniment place ever greater demands on personnel resources. Another factor augmenting social isolation since 2020 has been the COVID-19 pandemic, which reduced the number of in-person meetings between mental health service employees and service users, leading to fewer opportunities for social skills training.

While VR technology is explored in supporting well-being of older adults in long-term care homes,^5,6^ relatively limited research has addressed its use for social skills training^7^ with the exception of social skill training for individuals with autism spectrum disorder.^8–10^ Considerable innovation is needed to develop and test individual and societal interventions to reduce the number of people living in social isolation.^11^

Social isolation, which is defined as ‘an objective lack of interactions with others or the wider community,’ is linked to worse health outcomes.^12^ There are consistent evidence linking social isolation to depression, poor sleep quality, impaired executive function and accelerated cognitive decline^13^ and cardio-vascular disease.^14^ Although social isolation and loneliness are increasingly studied among older adults,^15^ few studies have been conducted among people with mental illnesses and suggestions on how to reduce social isolation and loneliness are rare.^16^

Comorbid social anxiety disorder is often found among people with severe mental illness, such as schizophrenia,^17^ general psychotic disorders,^18^ major depression and bipolar disorder,^19^ posttraumatic stress disorder^20^ and comorbid mental illness and substance use disorder.^21,22^ Social anxiety disorder requires special attention when studying social isolation because it involves a long-term overwhelming fear of social situations.^23^ Those with severe social anxiety often feel overly worried before, during and after social situations, and a core feature of the disorder is avoidance of social situations.^24^ Regardless of the reason for long-term social isolation, most mental health service users who withdraw from social situations benefit from some form of social skills training.

Loneliness and social isolation is identified as primary unmet needs among persons suffering from severe mental illness,^25^ and social skills training is recommended in recovery and reduction of disability due to mental illness^26^ and preferably in a community mental health setting.^27^ Those with severe mental illness often have a small or no network of friends and limited contact with their family.^28^ Mental health service employees are often the only people with whom some service users interact. Although many service users have previously received specialist mental health services treatment, this was either ineffective or not directed towards improving the social skills that would enable them to overcome their social isolation. Social problems still receive limited attention from specialist health services, which focus more on physical and mental health problems.^11^

Mental illnesses are caused and influenced by a complex interaction among congenital, biological, environmental and social factors, and they are rarely cured or managed by medical treatment alone.^29^ Thus, mental health services must work in a coordinated manner to address a range of social, psychological and medical care needs.^30^ Mental health treatment priority strategies are changing. Illness severity is no longer the only factor considered when assessing the need for specialist mental health treatment. Cost-effective models in current use may risk prioritising the many with less severe illness at the expense of the few with severe illness.^31^ Many with severe mental illness who previously received long-term treatment in specialist mental health services have no current contact with specialist mental health services, despite the severity of their mental illness. Thus, in many cases, the municipalities are the sole provider of health and welfare services. This suggests that new methods are needed to reach those with severe mental illness where they live, rather than through institutional treatment. The long-term shift from hospital-based treatment to local municipal mental health services implies that municipalities must have a role in mental health service innovation.^32^

Because the municipalities now have long-term responsibility for those with severe mental illness, the social skills training programme described herein was developed to equip these service providers with new tools to assist those experiencing social isolation. Our vision was to develop a programme to be delivered by municipal mental health service employees that would have a real impact on service users’ everyday lives. We used an exploratory approach, including lessons learned and recommendations for implementing useful technology in municipal mental health services. These lessons include to use collaborative research to lay the groundwork for effect studies of the developed programme in the municipal context. We also provide general suggestions for mental health services that are considering adopting this programme or developing something similar, for building competence in using VR as a mental health service tool.

## Methods

### Setting

Norwegian mental health services operate at the municipal level (i.e., primary health care) and the specialist level. Municipal-level responsibilities include prevention of health and social problems, assessment of functional abilities, early intervention and rehabilitation, follow-up, psychosocial support and counselling and referral to specialist services. Norway is currently divided into 356 municipalities, more than half of which have fewer than 5000 inhabitants and nearly 40% of which have fewer than 3000 inhabitants.

The municipality in which this study was set provides a broad service spectrum for those with mental health problems (e.g., housing services, mental health services for older adults, family support, various therapeutic services). Those with moderate or severe mental illness and extensive mental health care needs can contact the Health and Welfare Office, which then assesses the need for services and makes decisions regarding their provision. Simultaneously, those experiencing mild or common mental health problems can directly contact the different services without involving the Health and Welfare Office. Herein, we address aspects of the health services directed to those who gain approval for their use according to the Health and Care Services Act.

In the field of mental health, this municipality works towards a recovery-oriented approach where the services adapt to the service user’s perspective, whose wants, and needs are considered important in all activities of the services. About 70 per cent of the municipalities in Norway claim that they follow a recovery-oriented approach in their mental health services.^33^

### Development process

In 2018, we established a research collaboration among municipal mental health services employees, psychologists at a local firm, Coperiosenteret AS (which provided VR training) and mental health services researchers from SINTEF. The municipality involved in the research collaboration is the largest in mid-Norway and the third largest in Norway. This municipality’s mental health services cover a wide range, as described above.

During the past 20 years, SINTEF researchers have performed annual data collection in all municipalities regarding mental health services development. Through this data base, we have followed the development of resource use in the municipalities in services aimed at people with mental health problems and/or substance abuse problems measured by labour-years, and changes in the composition of expertise in the services. At the national level, mental health services have expanded from a few hundred labour-years at the end of the 1990s to more than 17,000 labour-years in 2022.^33^ The Directorate of Health receives annual research reports, which is used in further development of the sector.

In November 2018, the research team organised a VR workshop to which municipal mental health service employees, district psychiatric centre representatives and private firm psychologists with experiences with VR were invited. The workshop aim was to identify potential uses for VR technology in mental health services. According to the participants, the greatest potential for VR technology use was helping isolated individuals who need training in social skills and everyday activities, to allow them more active social lives.^34^

The clinical psychologist, the mental health professionals and the initial codeveloper together developed the prototype that was tested and adjusted based on input from two additional codevelopers. The resulting social skills training programme was developed with a combination of therapy, psychoeducation, VR social skills training and real-life social skills training.

### User Involvement

To develop the social skills training programme, one long-term service user was first recruited to work closely with the psychologists and mental health professionals employed by the municipal mental health services. This first codeveloper had lived a socially isolated life for many years and had received three rounds of treatment from the specialist mental health services, without significant improvement. Importantly, these treatments had not helped him overcome social isolation. He remained deeply troubled by social anxiety to the extent that he could not walk down the street or visit a grocery store without experiencing high levels of distress. In addition to being highly treatment motivated, he was also interested in technology, especially VR, having privately owned VR equipment and being familiar with VR scenarios and programmes. The service user attended 25 VR-based training sessions that combined psychoeducation and social skills training.

After drafting the first programme version, two additional service users were recruited to serve as codevelopers to test the programme prototype. They each attended 10 training sessions, after which further programme adjustments were made based on their feedback. The two additional codevelopers were not familiar with VR technology as the initial codeveloper, however fewer sessions were needed because the main content was established in the prototype.

### Technological expertise

The clinical psychologist leading the programme prototype development (ML) had previous experience treating severe mental illness through the specialist mental health services and was currently employed by the private VR training firm. ML developed the programme in collaboration with the service users and municipal mental health services employees. Experience and technical knowledge were shared with the municipality throughout the project. The three researchers were a psychologist with VR experience, a physiologist, and a mental health service researcher.

During the exploration period, researchers with expertise in VR, eye tracking and audio technologies were also involved. However, even though more advanced technological equipment was tested, in the end we decided to start with the simplest available equipment, that is, a cardboard headset combined with a smartphone. In the pilot study we will use Oculus Quest 2.

### Ethics

The research project was approved by the regional Research Ethics Committee in mid-Norway in January 2020 (REK 66949/2020). The researchers interacted freely with the mental health services employees and the psychologist who developed the prototype, but not with the three codeveloping service users (i.e., the service users remained anonymous to the researchers). The three involved service users provided written consent to participate in the study.

## Results

What follows is a description of the program that is developed, and not the results from piloting of the program, which will be the next step. The principles used in the programme are based on the well known cognitive model of social phobia.^35^ The resulting eight-step programme and examples of the service users’ problems and needs are described. Of note, some municipal mental health services professionals use the term ‘patient’ while others use ‘service user’. Herein, we use the latter term to emphasise application to the municipal mental health services rather than specialist mental health services.

### New service user assessment and research programme enrolment

The initial meeting with an individual referred for a mental health service was used to determine whether they were a candidate for enrolment in the social skills training programme. The inclusion criteria were mental illness, long-term social isolation, social anxiety, and interest in attempting to use a novel method under development. The exclusion criteria were heavy substance use during the past 2 months and currently expressing suicidal ideation.

**Figure 1.**
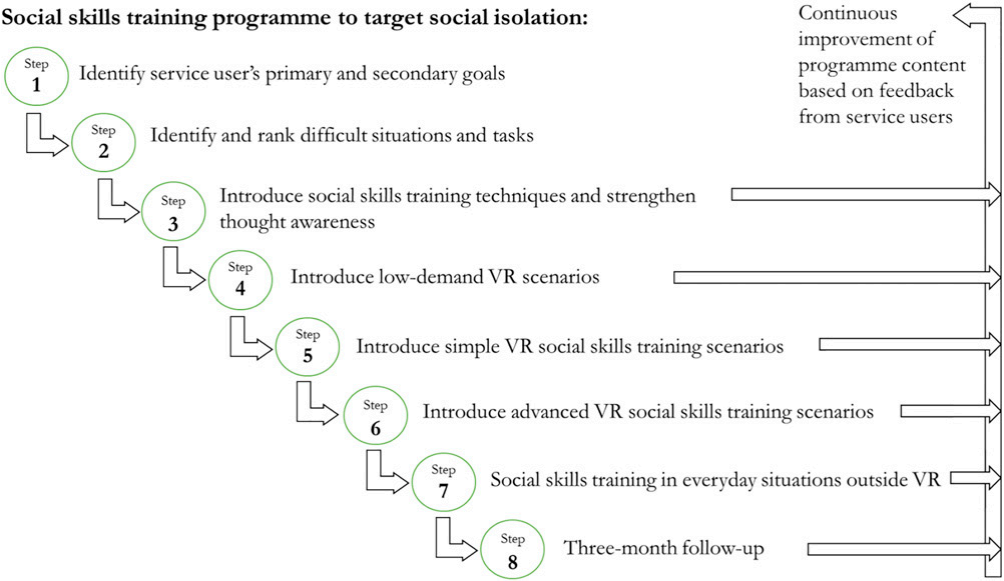
The 8-Step Social skills training programme to target social isolation.

In this programme development project and its future use, it is important to clarify service users’ expectations when discussing their eligibility for this programme. It should be clear that the service users’ efforts are essential and that the programme is not a ‘quick fix’. Specifically, it is stepwise training in social skills with increasing degrees of difficulty and includes professional assistance. The eight programme steps are explained next (Figure 1).

### Step 1: Identify service user’s primary and secondary goals

Rather than a thorough psychological assessment using different screening instruments, the first step in this programme is an informal conversation with the service user to identify their primary and secondary goals. This is achieved by simply asking them what they want to accomplish to improve their situation, an approach in line with the recovery model that emphasises and supports a person’s potential for recovery.

The primary goals of one service user who was a codeveloper were to have a girlfriend, eventually start a family and start practising a sport. These goals were discussed, and secondary goals were formulated: visit and make purchases at the grocery store and other shops, visit the gym without high anxiety levels, answer the phone and leave the apartment to take a daily walk. This service user had struggled with long-term social isolation and had few friends and limited family contact. He often assumed that people were staring at him from windows as he passed and that car drivers and passengers who passed him thought he was ugly and repellent. He had low self-confidence and considered himself a complete failure.

Other service users’ primary goals were similar: making new friends, expanding their social network, moving out of their parents’ house, and getting or continuing a job. Their secondary goals included attempting new activities, finding like-minded people, asking for something at a store, calling friends and mastering conversations with acquaintances or strangers.

### Step 2: Identify and rank difficult situations and tasks

In training service users to perform difficult tasks of daily life, it is necessary to identify specific difficult situations and activities. Talking to strangers, walking down the street, visiting the gym, and making new friends were difficult for these service users, but were ranked differently by service users according to their level of difficulty performing them.

### Step 3: Introduce social skills training techniques and strengthen thought awareness

Four sessions presenting the theoretical basis of social skills training are conducted using a simple flip chart or the like. Depending on the social problems described in Step 1 and their rankings in Step 2, education, and tips on how to handle them are provided in Step 3. A theoretical framing of social anxiety disorder involves education and information so that service users can better understand and cope with their illness. The four thematic sessions are:

**Session 1:** The goal is for the service user to learn how to challenge their beliefs. For instance, to question whether they know what others think of them (i.e., cognitive bias or negative automatic thoughts that lead to unreasonable, strong feelings). This involves identifying such thoughts, reflecting on them, and finding constructive alternatives; that is, learning how to challenge the thoughts that arise in situations that promote anxiety and fear.
**Session 2:** The goal is for the service user to differentiate between the foreground and background. People are usually unaware of their tendency to focus on signs around them for confirmation of their own hypotheses. If they believe that those around them have negative thoughts about them, they search for signs that confirm this. This strategy reinforces the negative automatic thoughts. One must look further outwards to actively seek that which weakens the hypothesis or belief.
**Session 3:** The goal is for the service user to understand the principles of anxiety and exposure, so they can cope with triggering situations. This means understanding the physiological reactions that are caused by fear and insecurity, or the hypersensitive social anxiety ‘alarm system’, which overrides logical reasoning that no danger exists. Because this system can learn with experience, it adapts to triggering situations but can also be recalibrated to make the situation more manageable. Anxiety symptoms usually follow a bell-shaped curve with exposure to a triggering situation, with a peak and then decline. Service users will through this session prepare for discomfort during training, which is necessary for improvement.
**Session 4:** The goal is to provide practical input for Step 5. For example, tips on how to initiate, continue and end a conversation are provided, including small talk as an introduction to deeper conversation. The focus is on starting a conversation with a general or open-ended comment to which anyone can respond (e.g., ‘The weather is nice today’) or soliciting an opinion/comment on a specific topic (e.g., ‘Did you see that film?’). The importance of validating the conversation partner’s perspective is also emphasised. Those with severe anxiety and negative automatic thoughts tend to appear stern, which can be off-putting to conversation partners, causing them to respond with negative signals, which trigger greater anxiety. One way to overcome this cycle is for the service user to encourage the conversation partner with ‘yes?’, nodding or repeating a key word. Finally, tips on how to end a conversation are presented, to prepare the service user with a plausible excuse for ending the conversation (e.g., if their anxiety level rises). Ending a conversation with a sense of achievement is important for service users, to break the cycle of serial, unsuccessful social interactions. Preparing backup topics like ‘What types of music do you like?’, ‘Do you like to do any types of sport?’ and ‘What is your favourite food?’ is also covered. This session ends with a discussion of how these strategies can be used in different situations and how to incorporate them into daily activities, which they must be committed to if their goal is to be more socially active. Each time the strategies are applied, the service user’s anxiety level is expected to decline. These theory-based topics were reinforced during Steps 5–7.

### Step 4: Introduce low-demand virtual reality scenarios

This step familiarises the service user with VR and introduces them to freely available YouTube videos. For example, for those who are afraid of heights, noises, snakes or visiting a store—or who simply want a new experience—the YouTube app can be used to view videos in 180° or 360° VR. Of course, this step can be skipped with service users familiar with VR.

### Step 5: Introduce simple virtual reality social skills training scenarios

The VR scenarios used herein include three social skills training elements:

1. Challenge the service user’s presumptions about what others think and shifting automatic negative thoughts from the foreground to the background.
2. Shift from searching for signs that confirm one’s presumptions to searching for signs that invalidate them.
3. Speaking with VR strangers using two simple task scenarios:
  • Scenario A: Three people (i.e., videos of real—not animated—people) sitting around a table in a work setting with the service user as the fourth person.
  • Scenario B: Ten people sitting around a long table taking notes, nodding, etc., while the service user stands and speaks at the short end of the table.
  • In both scenarios, pre-programmed tasks appear as single word instructions on a screen in front of the service user at three difficulty levels, from easiest to most difficult:

**Level 1: Association exercise.** A random word (e.g., ‘bicycle’, ‘snowflakes’, ‘dog’) appears on the screen every 5 seconds. For this association exercise, the service user says aloud whatever comes to mind in response to the word. This training level goal is to enable the service user to ignore their inner critic that prevents them from being socially active. When this level is mastered in both VR scenarios without high anxiety symptoms, the next level is introduced.

**Level 2: Easy storytelling exercise.** For these slightly more challenging tasks, questions appear (e.g., ‘What is your favourite month of the year?’, ‘What did you eat earlier today?’, ‘What is your favourite sports activity?’) to which the service user must respond with more than one word. The service user must think and focus on their answer, rather than their inner critic that activates negative automatic thoughts. A new question appears every 30 s.

**Level 3: Personal storytelling exercise.** For these more difficult, personal tasks, prompts appear on the VR screen (e.g., ‘Tell me about your first day at school’, ‘Tell me about why you like summer’). Like in Level 2, a new topic appears every 30 s, but in this level the participant must tell a story.

Each level is usually considered most difficult with the service user’s first attempt, after which they become gradually easier. When no discomfort occurs during Level 3, Step 6 is initiated.

Of note, individual variation in duration to mastery of these exercises is expected. During programme development, we learned that some service users felt that the work setting scenario was not relevant for them. Based on this, we experimented with creating new VR videos in local outdoor environments. These were low-threshold settings in which the researchers’ family members were actors sitting on a bench eating the packed lunch that Norwegians often carry for walking tours. Long-term, it may be possible to film personalised scenes for individual service users. Creating short VR 360° videos does not require specific technical training.

A simplification of Step 5 is to do this without using VR. Then the tasks in Level 1–3 are presented orally to the service user. We made VR scenarios where the tasks were integrated in a VR film. This might not be necessary.

### Step 6: Introduce advanced virtual reality social skills training scenarios

In this step, virtual rooms with actual people are introduced. The degree of difficulty is high, with service users required to approach and speak to strangers. We used vTime XR and Bigscreen technology. vTime XR is a free-to-play VR and augmented reality social network in which up to four people can meet and speak in a private chat room in real time. Users can customise an avatar and select a three-dimensional environment to host the chat. Bigscreen is a free VR operating system with which it is possible to watch movies and meet friends. Other platforms could be used for this step, that is, the crucial element is to meet and talk with strangers in a virtual setting.

This real-time setting in which service users meet strangers includes many elements that can cause anxiety symptoms. At first, the municipal mental health services employees joined the participants in a virtual room, creating a safe environment for the service users. After this adaption process, the service users were able to attend virtual meetings alone and practice speaking with strangers until they felt comfortable.

### Step 7: Social skills training in everyday situations

Now social skills training in everyday life (i.e., outside VR) begins. This tailored training reinforces Steps 1 and 2, with mental health service employees supporting the service user throughout. It is expected that training in everyday life will be easier and more effective after VR training because the service user has already been exposed to anxiety triggers in a safe, controlled environment.

### Step 8: Three-month follow-up

Many service users will continue with the same or other municipal mental health services after completing this programme and are thus available for follow-up consultation to gather data on the programme’s effects. This information can be used for continuous improvement of the content of the programme.

### Need for flexibility

Based on experiences during the development and feedback from the codevelopers, it is important that the programme is flexible and can be adjusted according to the needs of individual service user. Different and freely available VR scenarios can easily be found online according to the difficult situations identified in Step 2, and personalised films may be recorded locally. Though all steps should be covered, two or more steps can be combined within a session. Steps could also be divided in several smaller steps when needed. Our goal was to develop a programme suitable for those with severe mental illness; however, service users with less severe mental health problems may also benefit from VR with or without working through all eight steps. The length of each session will vary according to the needs and preferences of the service user. Some will prefer only a few minutes in VR each time, while others can continue for several hours.

### Educating municipal mental health professionals to support service users with virtual reality technology

To implement the VR technology in the mental health services and thus make the technology available to service users, the professional mental health workers must be familiar and comfortable with the technology. The researchers developed and delivered a one session practical VR course to 35 mental health professionals employed in the municipal mental health services. The course, technical equipment used, and experiences from the course are described in a separate article.^1^

## Discussion

Social isolation increases the risk of deteriorating mental and physical health; thus, social skills training could become an essential preventive measure for service users with severe mental illness. Herein, a social skills programme using VR technology was developed through a collaboration among service users (with varying levels of mental illness severity and social isolation), municipal mental health services professionals, mental health service researchers and a local firm providing VR training. The resulting programme is comprised of eight steps: 1) identify service user’s primary and secondary goals; 2) identify and rank difficult situations and tasks; 3) introduce social skills training techniques and strengthen thought awareness; 4) introduce low-demand VR scenarios; 5) introduce simple VR social skills training scenarios; 6) introduce advanced VR social skills training scenarios; 7) social skills training in everyday situations outside VR; and 8) three-month follow-up.

We suggest that incorporating VR steps in social skills training can reduce the time to implementing skills in the real world. VR provides opportunities to practise social skills, even among users with high anxiety levels. This process cannot typically occur in standard therapy as it is difficult to manage triggering social anxiety in an office setting. VR technology can get service users into situations that trigger social anxiety without needing to experience the actual situation.

### From medical focus to recovery in mental health services

Nurses, social workers, and other municipal mental health services employees all provide services for those with severe mental illness, but these services have received limited research attention compared with the specialist mental health services. However, there are differences between specialist and municipal health services. Medical treatment and then recovery is inconsistent with a recovery approach in which service users express and construct the goals they want to achieve with assistance from professionals, whose role is to encourage the service user to set new goals and live their lives, perform activities and develop meaningful relationships.^36^ Therefore, municipal services may be better equipped than specialist mental health services to develop, test and implement social skills training programmes to assist those with severe mental illness to overcome social isolation.

### Medication-free treatment

An increasing medication-free focus to reduce high levels of coercive treatment has recently occurred in Norway. Following pressure from mental health service user organisations in 2015, the Norwegian Ministry of Health and Care determined that medication-free services should be provided in all four health regions. In 2018, medication-free treatment was offered in 25 Norwegian locations.^37^ A recent study revealed that experiencing negative medication effects was among the important reasons for patients wanting medication-free treatment, and that alternatives to medication were generally unavailable in ordinary health care services.^38^ Greater medication-free treatment alternatives would strengthen the recovery focus in mental health services, and using the technology described herein in such settings might allow more service choices for those with severe mental illness.

### Lower cost and easier to use technology

Municipalities and other public services cannot be expected to prioritise large investments in VR equipment without evidence of its benefits. Although the price of VR equipment has dropped considerably in recent years, advanced equipment is still expensive. Thus, considering affordable alternatives for programme development, piloting and implementation is essential. A recent pain study showed that inexpensive VR devices may be a safe, portable and cost-effective method for altering pain perception and improving tolerance.^39^ We opted to use the Cardboard headset and smartphone. A primary reason for this is that more advanced headsets require technical assistance and thus more people touching the equipment during the pandemic (with subsequent equipment sterilisation requiring considerable resources), whereas the Cardboard headset is touched only by the user. Low cost is another important consideration in the public services, where changing priorities often means fewer resources for other vulnerable populations.

### More focus on physical and mental health than on social problems in health services

Although social isolation can have major health consequences, social problems receive limited attention from the specialist health services.^11^ Social work is usually conducted locally, by primary services. Thus, municipalities’ health and social workers should be at the frontline for developing effective training programmes to prevent social isolation-related health problems. Programme development similar to that described herein should also be conducted in close collaboration with the primary health care services. However, it has also been suggested that specialist mental health services should also focus on social isolation, especially in suicide risk assessment.^40^

### Implementation status

The municipal mental health service involved in this project, was introduced to VR at the first workshop we had, back in 2018. They received VR equipment as part of the project plan, and 35 mental health professionals completed a practical low-cost introductory VR course developed and held by the researchers. One or two mental health professionals participated in every session together with the codeveloping service users and the psychologist in the development phase of the programme, and they now conduct the pilot study without the psychologist who developed the programme. Researchers follow the pilot by interviewing mental health professionals regularly to collect their experiences with using the programme, however the researchers are not involved in the delivery of the programme to the individual service user. The service has organised a dedicated VR-team, and internalised the competence developed through the project, i.e., they have accumulated the knowledge of how to use VR technology in the provision of mental health services throughout the project.

### Need for more research

The work described herein is the first step in developing an effective social skills training programme using VR technology in a primary mental health setting. The next step will be to test its effects on social isolation at the individual level.

## Conclusion

Reducing the number of people living in social isolation will require significant future research efforts and innovations. The programme developed is intended for those with long-term mental illnesses, living social isolated lives. Given the number of municipal mental health services users with social anxiety-related problems, such development may ultimately make a meaningful impact. Our goal was to provide a tool for municipal mental health professionals, to assist their effort to help service users out of social isolation. As a next step, we will now pilot this programme in the municipality among 10 mental health service users.

## Note

1 Unpublished data.

## Appendix

### Abbreviations

VR: Virtual reality
